# Expression Profiling of the Slow Rusting Resistance Genes *Lr34*/*Yr18* and *Lr67*/*Yr46* in Common Wheat (*Triticum aestivum* L.) and Associated miRNAs Patterns

**DOI:** 10.3390/genes14071376

**Published:** 2023-06-29

**Authors:** Julia Spychała, Agnieszka Tomkowiak, Aleksandra Noweiska, Roksana Bobrowska, Jan Bocianowski, Michał Książkiewicz, Aleksandra Sobiech, Michał Tomasz Kwiatek

**Affiliations:** 1 Department of Genetics and Plant Breeding, Poznań University of Life Sciences, 11 Dojazd Str., 60-632 Poznań, Poland; julia.spychala@up.poznan.pl (J.S.); aleksandra.noweiska@up.poznan.pl (A.N.); roksana.bobrowska@up.poznan.pl (R.B.); aleksandra.sobiech@up.poznan.pl (A.S.); michal.kwiatek@up.poznan.pl (M.T.K.); 2 Department of Mathematical and Statistical Methods, Poznań University of Life Sciences, 28 Wojska Polskiego St., 60-637 Poznań, Poland; jan.bocianowski@up.poznan.pl; 3 Institute of Plant Genetics, Polish Academy of Sciences, Strzeszyńska 34, 60-479 Poznań, Poland; mksi@igr.poznan.pl

**Keywords:** leaf rust, APR resistance, slow rusting, microRNA, RT-qPCR, ddPCR

## Abstract

The main efforts in common wheat (*Triticum aestivum* L.) breeding focus on yield, grain quality, and resistance to biotic and abiotic stresses. One of the major threats affecting global wheat cultivation and causing significant crop production losses are rust diseases, including leaf rust caused by a biotrophic fungus *Puccinia triticina* Eriks. Genetically determined resistance to leaf rust has been characterized in young plants (seedling resistance) as well as in plants at the adult plant stage. At the seedling stage, resistance is controlled vertically by major R genes, conferring a race-specific response that is highly effective but usually short-lived due to the rapid evolution of potentially virulent fungi. In mature plants, horizontal adult plant resistance (APR) was described, which provides long-term protection against multiple races of pathogens. A better understanding of molecular mechanisms underlying the function of APR genes would enable the development of new strategies for resistance breeding in wheat. Therefore, in the present study we focused on early transcriptomic responses of two major wheat APR genes, *Lr34* and *Lr67,* and three complementary miRNAs, tae-miR9653b, tae-miR9773 and tae-miR9677b, to inoculation with *P. triticina*. Plant material consisted of five wheat reference varieties, Artigas, NP846, Glenlea, Lerma Rojo and TX89D6435, containing the *Lr34*/*Yr18* and *Lr67*/*Yr46* resistance genes. Biotic stress was induced by inoculation with fungal spores under controlled conditions in a phytotron. Plant material consisted of leaf tissue sampled before inoculation as well as 6, 12, 24 and 48 h postinoculation (hpi). The APR gene expression was quantified using real-time PCR with two reference genes, whereas miRNA was quantified using droplet digital PCR. This paper describes the resistance response of APR genes to inoculation with races of leaf rust-causing fungi that occur in central Europe. The study revealed high variability of expression profiles between varieties and time-points, with the prevalence of downregulation for APR genes and upregulation for miRNAs during the development of an early defense response. Nevertheless, despite the downregulation initially observed, the expression of *Lr34* and *Lr67* genes in studied cultivars was significantly higher than in a control line carrying wild (susceptible) alleles.

## 1. Introduction

Leaf (brown) rust represents one of the world’s greatest threats to wheat production. The disease is caused by the pathogenic fungus *Puccinia triticina* Eriks. The appearance of the disease is observed globally, particularly in regions where common wheat is grown intensively, including Europe, Australia, China and North America [1,2]. The *P. triticina* fungus is a representative of the order *Pucciniales* (formerly *Uredinales*). All representatives of the order *Pucciniales* are absolute parasitic pathogens of vascular plants with a rather complicated life cycle. In addition to common wheat, the pathogen is capable of infecting other cereals, i.e., rye (*Secale cereale*), barley (*Hordeum vulgare* L.) and triticale (*×Triticosecale)*. Globally, under optimal conditions, the disease can cause a significant yield decrease of up to 40% [3,4]. Infection occurs with the aid of wind, which carries the spores to the host plants. Optimal conditions for the development of infection are between 15 and 20 °C, although disease emergence is also possible in a wider temperature range of 2 to 35 °C. A factor particularly conducive to infection is high humidity. Under favorable environmental conditions, urediniospores emerge 7–10 days after infection [5,6,7].

The selection of varieties with favorable traits, such as high yield potential and desirable nutritional quality, requires also improved resistance to pathogenic fungi. The understanding of molecular mechanisms underlying plant–pathogen interactions will enable the development of new strategies for resistance breeding in wheat. Genetically determined resistance to leaf rust has been characterized both in young plants (seedling resistance) and in plants at the mature stage. At the seedling stage, resistance is controlled vertically by R major genes, which are frequently broken by coevolving fungal pathogens. In mature rust-resistant plants, horizontal adult plant resistance (APR) is observed, which is characterized by better durability. The occurrence of non-race-specific resistance is often manifested as a *slow rust* effect, which is associated with slower infection development, a longer latency period and the formation of fewer uredinia. APR provides partial but durable resistance to the pathogen [8]. However, most *Lr* genes confer race-specific resistance, consistent with the “gene-for-gene” concept [9]. In total, approximately 80 leaf rust resistance genes have been identified and characterized in bread wheat, durum wheat species and diploid wheat [10]. The distribution of resistance genes in the wheat genome is very dispersed, as *Lr* genes were found on 20 of the 21 chromosome pairs present in a hexaploidy wheat. A collection of different enzymatic markers and PCR-based molecular and microsatellite markers have been used to identify these genes [1]. Although many rust resistance genes have been identified, the main problem is short-term effectiveness due to the rapid occurrence of new pathotypes capable of breaking resistance. To date, at least four effective genes have been identified: *Lr34*/*Yr18*/*Pm38*, *Lr46*/*Yr29*/*Pm39*, *Sr2*/*Lr27*/*Yr30* and *Lr67*/*Yr46*/*Pm46*, which conferred partial but durable long-term resistance. This type of resistance determines the slow rusting effect and contributes to reducing the rate of disease development. The advantage of using varieties carrying this type of genes in resistance breeding is the pleiotropic effect of their expression, which contributes to an increase in the level of resistance due to intergene interaction and immunization against other wheat diseases such as yellow rust (*Puccinia striiformis* Westend.) and powdery mildew caused by *Blumeria graminis* (DC.) Speer [11]. At present, only APR genes such as *Lr34*, *Lr46* and *Lr67* are significantly exploited in resistance breeding, because they confer consistent and long-lasting resistance to diverse fungal pathotypes found under different climatic and environmental conditions. Recent scientific reports indicate that rust resistance, which results from a combination of genes conferring race-specific resistance and genes that are nonspecific to a particular race of the pathogen, is the more durable and most preferred resistance in cultivated wheat varieties [2,5].

The *Lr34* gene was first described in 1977 in Canada in wheat line PI58548 [12]. Later studies showed that it is also present in many other wheat varieties. Further analysis of the *Lr34* gene revealed its location in the genome, so that it was shown to be located on wheat chromosome 7DS. The *Lr34* gene was first identified in the “Frontana” variety [13]. The *Lr34*/*Yr18*/*Pm38* gene complex confers partial resistance to leaf rust, yellow rust and powdery mildew, respectively. In addition, the locus is associated with tolerance to stem rust (*Puccinia graminis* Pers.) and barley yellow dwarf virus (*Bdv1*) [8,14]. The *Lr34* is the most well-studied APR-type gene and is also being used as a model to study the molecular basis of the persistent resistance phenomenon [6,15]. The *Lr34* gene is effective at the adult plant stage and, under favorable conditions, at the flag leaf stage. *Lr34* gene expression was higher at lower temperatures of 13–17 °C than at 25–30 °C [16,17]. Unfortunately, resistance conditioned by gene *Lr34* is less effective at high temperatures.

The length of the *Lr34* gene is 11,805 base pairs (bp). The *Lr34* gene encodes the membrane protein ABC transporter, consisting of 1401 amino acids (24 exons). The gene has been cloned and two allelic variants have been distinguished in it: susceptible (*Lr34sus*) and resistant (*Lr34res*) [18,19]. These alleles differ in by 3-nucleotide indel resulting in a deletion of phenylalanine (Phe) at position 546 (exon 11) and a single nucleotide polymorphism (SNP) resulting in a tyrosine (Tyr) to histidine (His) substitution at position 634 (exon 12) in the Lr34Res protein [20,21]. Moreover, additional A/T SNP polymorphisms were discovered in intron 4 and a 1-nucleotide indel in exon 10 that causes a frameshift [18].

The *Lr67* gene was identified in the common wheat cultivar “PI 250413” and transferred to the cultivar “Thatcher” to produce the line “RL6077”. The *Lr67* gene also shows pleiotropic effects on stem rust and yellow rust, but with a lower effect on leaf rust resistance than *Lr34*. The *Lr67*/*Yr46*/*Pm46* gene complex has been mapped to chromosome arm 4DL. The *Lr67* gene encodes a hexose transporter in two allelic variants, with resistant—the LR67res protein differing from the susceptible form LR67sus by two amino acids, leucine (Leu) and arginine (Arg). Both allelic variants have identical promoter regions. The *Lr67* gene has homeologous genes on common wheat chromosomes 4A and 4B [8,22].

Interestingly, both *Lr34* and *Lr67* genes also induce premature aging of mature leaf tips (leaf tip necrosis) suggesting mechanistic commonality. Consistent with this hypothesis is the lack of additivity of these two resistance genes for adult plants. However, the products of these two genes are different. *Lr34* encodes an ABC transporter protein that is suggested to transport abscisic acid while *Lr67* encodes a hexose transporter protein no longer capable of sugar transport. The manner in which these two genes provide resistance has not yet been fully established; however, a remarkable feature of *Lr34* is its durability having been used in agriculture for many decades without being overcome [8].

Modern breeding technologies and biotechnology strategies are valuable tools to create climate-resilient crops. In addition, to understand plant responses against various abiotic and biotic stresses, research efforts have been undertaken to reveal the genetic basis underlying resistance/tolerance mechanisms [2,23]. Race-specific disease resistance is based on rapid recognition of invading pathogens and effective activation of plant–host defense mechanisms. At the onset of disease, the hypersensitive response (HR) limits the spread of the pathogen and activates defense mechanisms in the uninfected part of the plant. Considerable progress has been made in understanding the signaling process involved in several plant pathogen interactions. However, there is little research on the interaction of fungal pathogens infecting wheat during the early stages of nonrace-specific APR defense response. Upon infection, expression of resistance genes can be also regulated by endogenous small noncoding miRNAs [7]. Plant miRNAs are small molecules of approximately 21 nucleotides that complementary bind to target mRNAs and repress gene expression by targeting the mRNA for degradation or by inhibiting translation. Plant studies have revealed the key role of miRNAs in various regulatory pathways involving almost all biological processes, including auxin signaling, meristem formation, leaf formation, lateral root formation, reproduction, and defense responses to biotic and abiotic stresses [24,25].

The significant role of slow rusting resistance genes *Lr34*/*Yr18* and *Lr67*/*Yr46* in global common wheat breeding contrasts with the relatively limited knowledge of the early stages of APR defense response deployment. Therefore, in the present study, we focused on transcriptomic profiling of these genes and candidate complementary miRNAs in a timeline spanning the first 48 h postinoculation. Five reference wheat varieties (Artigas, NP846, Glenlea, Lerma Rojo and TX89D6435) carrying resistant alleles of studied genes were analyzed. The expression of *Lr34*/*Yr18* and *Lr67*/*Yr46* genes was quantified using real-time PCR, whereas the level of miRNA was evaluated using droplet digital PCR (ddPCR).

## 2. Materials and Methods

### 2.1. Plant Material and Leaf Rust Inoculation in Controlled Conditions

Reference common wheat varieties carrying the resistant alleles of *Lr34* and *Lr67* genes were obtained from the National Small Grains Collection of the National Plant Germplasm System (NPGS) of the United States Department of Agriculture—Agricultural Research Service (USDA-ARS), located at the Agriculture Research Station in Aberdeen, Idaho (USA). Four resistant spring varieties: NP846 (PI 322263), Glenlea (CItr 17272), Artigas (PI 192535), Lerma Rojo (Cltr 13651) and one winter variety TX89D6435 (PI 584759) were selected. The negative control in the experiment was the Artigas* (PI 73046) variety, which has no *Lr* resistance genes. Molecular analysis of the reference varieties using flanking markers confirmed the presence of resistant alleles of *Lr34* and *Lr67* genes. The spores of the fungus were collected from infected field experiments located in different parts of Poland, in localities where branches of Polish breeding companies are located: Smolice 52.1000° N 19.0500° E, Strzelce 53.0167° N 16.9667° E, Kobierzyce 50.9667° N 16.9167° E, Nagradowice 52.4167° N 16.9667° E. The inoculation experiment was established under controlled conditions in a phytotron. At the beginning of the experiment the temperature in the phytotron chamber was set at 18 °C and 16 °C during the day and night, respectively. Photoperiod 18 h, humidity in the range of 70–90%. The emission spectrum of a light source with a photon flux of 572 μE. After one month, the temperature was raised to 20 °C during the day and 17 °C at night. Biotic stress was induced at the adult plant stage (60 days from sowing) by spraying whole plants with a spore suspension at a concentration of approx. 5 × 10^5^ spores/mL (with Tween 20 reagent). The infectious material was a mixture of *P. triticina* isolates from different regions of Poland. The mixture was prepared immediately before inoculation. Leaf tissue fragments for molecular analyses were taken immediately before inoculation (0 h) and 6, 12, 24 and 48 h postinoculation (hpi). Three biological replicates per time period and genotype were sampled.

### 2.2. RNA Isolation and Synthesis of cDNA

Isolation of RNA from wheat leaves was carried out using the Maxwell^®^ RSC Plant RNA isolation Kit, according to the protocol provided by the manufacturer. RNA concentration was measured using a NanoDrop 2000 spectrophotometer (Thermo Fisher Scientific, Waltham, MA, USA). Synthesis of single-stranded cDNA was carried out using 1 µg of isolated RNA and iScript cDNA Synthesis Kit (Bio-Rad, Hercules, CA, USA). The temperature profile of the cDNA synthesis reaction was as follows: priming for 5 min at 25 °C, reverse transcription for 60 min at 46 °C, inactivation of reverse transcriptase for 1 min at 95 °C, cooling to 4 °C and storage at −20 °C.

### 2.3. Primer Design and Development of Standard Curves for Lr34 and Lr67 Genes

Primers for quantitative PCR were designed based on gene sequences (LOC123169079 for *Lr34*, MK425206 for *Lr67*) from the NCBI (https://www.ncbi.nlm.nih.gov/, accessed on 14 August 2019) and Ensembl Plants (https://plants.ensembl.org/Triticum_aestivum/Info/Index, accessed on 7 April 2021) databases using the Primer3Plus tool (https://www.bioinformatics.nl/cgi-bin/primer3plus/primer3plus.cgi, accessed on 1 January 2006). For both *Lr34* and *Lr67* genes three very similar duplicates were identified in common wheat genome (LOC123169079, LOC123087958, LOC123152986 and LOC123092952, LOC123098224, LOC123085159, respectively); therefore, universal primers were designed, amplifying studied genes together with other similar homologs (Table 1). For each primer pair (i.e., each gene), a PCR with gradient of annealing temperature in the range of 50–58 °C was performed to select optimal annealing temperature common for both analyzed genes. The matrix for this PCR was composed as an equalized mixture of cDNAs, derived from 12, 24 and 48 hpi RNA samples, to increase the likelihood of remarkable expression levels of resistance genes. GoTaq^®^ G2 Flexi DNA Polymerase (Promega, Madison, WI, USA) was used. The composition of the reaction mixture was as follows: PCR buffer—4 µL, dNTP (10 µM)—1.6 µL, nuclease-free water—10.1 µL, MgCl_2_ (25 mM)—1.6 µL, primers (10 µM)—0.5 µL each, polymerase (5 u/μL)—0.2 µL, cDNA mixture—1.5 µL. PCR profile was as follows: initial denaturation for 2 min at 94 °C, followed by 35 cycles including denaturation for 1 min at 95 °C, annealing of primers for 30 s in temperature gradient of 50–58 °C, extension for 30 s at 72 °C, final extension for 5 min at 72 °C and cooling to 4 °C. PCR products were separated by electrophoresis (10 V/cm) in a 2% agarose gel in Tris EDTA buffer pH 8.0 (Sigma-Aldrich, St. Louis, MO, USA) with SYBR Safe DNA staining (Invitrogen, Carlsbad, CA, USA) and visualized on UV-transilluminator (Uvitec, Thermo Fisher Scientific, Waltham, MA, USA). As a result of these analyses, optimal annealing temperature for quantitative PCR was selected (53.5 °C). Once the reaction conditions were established, five PCR runs in a volume of 20 µL each were performed to obtain a volume of 100 µL of the target amplicons. The amplification temperature profile was as follows: initial denaturation for 2 min at 94 °C, followed by 35 cycles, denaturation for 1 min at 95 °C, annealing of primers for 30 s at 53.5 °C, extension for 30 s at 72 °C and final extension for 5 min at 72 °C. Amplicon purification was carried out using QIAquick PCR Purification Kit (Qiagen, Hilden, Germany), according to the protocol provided by the manufacturer. Concentration of purified PCR products was measured using a NanoDrop 2000 spectrophotometer (Thermo Fisher Scientific). In order to make standard curves, a series of dilutions was prepared for each analyzed gene. Standard curves were developed following previously reported protocol [26].

### 2.4. Selection of Reference Genes for Quantitative PCR

**Figure 1:**
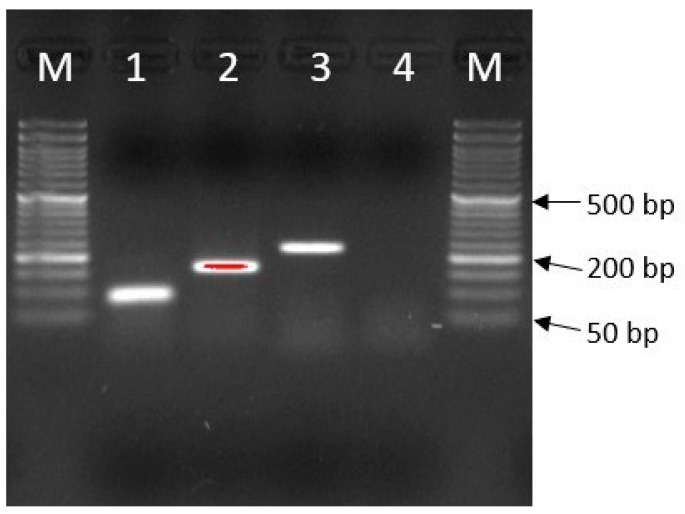
Agarose gel electrophoregram showing amplification results for reference genes; 1—*TUBβ*, 2—*ARF*, 3—*RLI*, 4—*EF2-1*, M—size marker; 50 bp DNA Ladder (NIPPON Genetics EUROPE GmbH).

Results of quantitative PCR are usually referred to the reference genes that are expected to have stable expression profile in a specific experimental setup. Based on the literature data, four common wheat genes were selected (Table 2) as candidate references: *TUBβ* (β-Tubulin, U76897), *ARF* (ADP-ribosylation factor, AB050957), *RLI* (Rnase L inhibitor-like protein, AK331207.1) and *EF2–1* (Elongation factor, XM_009218808) [27,28]. They were tested by performing PCR amplification on cDNA pooled array (samples collected at 12, 24 and 48 hpi), followed by electrophoresis (10 V/cm) in a 2% agarose gel (Figure 1, Table 2). At this stage, nonspecific products were obtained for the *RLI* gene after real-time PCR reactions, which was the cause of discarding the reference gene. The occurrence of nonspecific products was observed by analyzing melting curves. For the remaining candidate reference genes, a standard curve was developed using the same approach as for *Lr34* and *Lr67* genes (except for primer annealing temperature: 60 °C instead of 53.5 °C). After analysis of standard curves, two genes with the highest efficiency values and R^2^ > 0.98, *TUBβ* and *ARF*, were selected as references for quantitative PCR (Table 2).

### 2.5. Quantitative (Real-Time) PCR

Expression analysis using real-time PCR was performed in 96-well plates (Eppendorf, Hamburg, Germany) using iTaq Universal SYBR Green Supermix and CFX Connect Real-Time PCR Detection System (Bio-Rad). The composition of the qPCR reaction mixture was as follows: Supermix—5 µL, “forward” and “reverse” primers (10 µM)—0.5 µL each, nuclease-free water—3 µL and cDNA template—1 µL. In accordance with current MIQE recommendations for the validity of real-time PCR analyses, for every genotype/time points three biological replicates were used, and three technical replicates were prepared for each [29]. The qPCR reactions were performed with NTC (no template control), representing the reaction mixture itself, and with inter-run calibration sample (*ARF*), both were applied in three technical replicates. The following temperature profile was used for quantitative PCR: initial denaturation for 3 min at 95 °C; then, 40 cycles: denaturation for 10 s at 95 °C and annealing of primers for 30 s at 53.5 °C (*Lr34* and *Lr67* genes) or 60 °C (*TUBβ* and *ARF*) with fluorescence measurement. High resolution DNA melting was performed after PCR to control the specificity of amplification: melting temperature range 65–90 °C; every 5 s the temperature was increased by 0.5 °C and fluorescence measurement was taken. Calculations of ∆∆Cq included both reference genes using CFX Maestro (Bio-Rad).

### 2.6. Expression Analysis of miRNAs Using Droplet Digital PCR

In this study, the expression of three miRNAs (tae-miR9653b, tae-miR9773, tae-miR9677b) related to the *Lr34* was analyzed. Available databases for common wheat and literature data did not show miRNA molecules complementary to the *Lr67* gene. The coding sequence of the *Lr34* gene (TraesCS7D02G080300.1) was downloaded from the Ensembl.

Plants database for common wheat (https://plants.ensembl.org/Triticum_aestivum/Info/Index, accessed on 7 April 2021) was analyzed in the psRNAtarget database (http://plantgrn.noble.org/psRNATarget/, accessed on 1 January 2017). The “Submit Target Candidates” option was selected, which allowed analysis of the selected sequence and searching for related miRNAs. The sequences of the three miRNAs analyzed were found in the miRBase database (https://www.mirbase.org/, accessed on 5 June 2009) and, using the Integrated DNA Technologies (IDT) website (https://eu.idtdna.com/pages, accessed on 15 June 2012), stem-loop primers were designed for miRNA reverse transcription and ddPCR reactions according to the protocol [30,31]. The primer sequences intended for both reactions are shown in the listing (Table 3 and Table 4). The species *Triticum aestivum* was selected and the names of the selected miRNAs were searched. A mature sequence was required to design primers for complementary miRNAs, so the “Mature sequence” function was selected. The mirVana™ miRNA Isolation Kit with phenol (Thermo Fisher Scientific) was used to extract the RNA fraction containing miRNA, whereas the concentration and quality of isolates were measured spectrophotometrically using NanoDrop 2000 (Thermo Fisher Scientific). The concentration and quality of the isolated RNA was checked using a spectrophotometer (DeNovix, Wilmington, DE, USA) at 260 nm. A fixed amount of isolated RNA (1 µg sRNA and 1.5 µg total RNA) was reverse transcribed using SuperScript IV Reverse Transcriptase (Thermo Fisher Scientific), stem-loop primers and the following protocol: incubate for 30 min at 16 °C; 60 cycles at 30 °C for 30 s, 42 °C for 30 s and 50 °C for 1 s; and incubation at 85 °C for 5 min. To quantify the number of miRNA molecules in plant samples, a ddPCR mixture composed of 10 µL of ddPCR Super Mix Eva Green (Bio-Rad), primers (to reach final concentration of each primer 200 nM), template (reverse transcribed, elongated miRNA) and RNase-free H_2_O was used. A 20 µL reaction mixture was used to generate droplets in an 8-well cartridge placed in a QX100 droplet generator (Bio-Rad Laboratories, Inc., Hercules, CA, USA). The droplets were carefully transferred to a 96-well ddPCR plate and heat-sealed with foil (Bio-Rad). Then, cDNA was amplified in a T100 PCR thermal cycler (Bio-Rad) under the following cycling conditions: 5 min denaturation at 95 °C, followed by 40 cycles with a three-step thermal profile of 30 s denaturation at 95 °C, 30 s annealing at 58 °C and 45 s extension at 72 °C. Subsequently, the products were kept at 72 °C for 2 min for the final extension. After amplification, the products were cooled to 4 °C for 5 min, then heated to 90 °C for 5 min and finally cooled again to 12 °C. The droplets were quantified in a QX100 droplet reader (Bio-Rad). Data acquisition and analysis were performed using QuantaSoft software (Bio-Rad). Positive droplets containing amplification products were distinguished from negative droplets by setting the fluorescence amplitude threshold to the lowest value in the positive droplet cluster.

### 2.7. Preparation of Lr34 and Lr67 Amplicons for Sanger Sequencing

Sanger sequencing was performed to confirm that the desired amplicons were obtained for the *Lr34* and *Lr67* genes and to characterize the differences between the resistant genotypes and the susceptible genotype (Artigas* variety). For the *Lr34* and *Lr67* genes, 5 PCR reactions were performed for the corresponding primer pairs. The composition of the reaction mixture was as follows: nuclease-free H_2_O—10.1 µL; buffer—4 µL; dNTP (10 µM)—1.6 µL; MgCl_2_ (25 mM)—1.6 µL; primer F (10 µM)—0.5 µL; primer R (10 µM)—0.5 µL; polymerase (5 u/μL)—0.2 µL; and template—1.5 µL. GoTaq^®^ G2 Flexi DNA Polymerase reagents from Promega were used to prepare the PCR reaction. The following amplification temperature profile was used: initial denaturation for 2 min at 94 °C; followed by 35 cycles: denaturation for 1 min at 95 °C, primer annealing for 30 s at 60 °C, extension for 30 s at 72 °C; final extension for 5 min at 72 °C and cooling to 4 °C. Purification of the amplicons was performed using QIAquick PCR Purification Kit (Qiagen), according to the protocol provided by the manufacturer. After purification, the samples were resuspended in Elution Buffer in a volume of 30 µL. Sanger sequencing was performed by Genomed (Warsaw, Poland). Obtained results were processed using Geneious software.

### 2.8. Statistical Analysis

The Kolmogorov–Smirnov test was used to test the null hypothesis that a set of normalized expression data for a particular gene and line comes from a normal distribution. This test was passed for all gene × genotype combinations except *Lr34* in TX89D6435 and tae-miR9773 in NP846 (*p*-values 0.028 and 0.023, respectively). To assess the equality of variances, Brown–Forsythe variant of Levene’s test was calculated (passed for all tested time point × gene × genotype combinations). For comparison of means, a two-tailed t-Student was performed. The following comparisons were performed for *Lr34* and *Lr67* genes and studied miRNAs: (i) expression in each examined time point after inoculation vs. expression before inoculation and (ii) expression in each examined time point vs. expression in a correlated time point in Artigas line carrying susceptible allele. To test stability of reference genes, the following comparisons were performed: cycle threshold (cT) value in each time point after inoculation vs. cT value before inoculation (Supplementary Table S3). This test revealed significant increase in reference cT values in some comparisons, mostly at the beginning of experiment or in the middle time points, that could eventually result in increase in calculated expression of studied genes. However, in all those cases, expression of studied genes was not significantly increased; therefore, observed transient instability of reference genes did not contribute to false positives. For visualization of gene expression results, cluster analysis (UPGMA method) with the Euclidean distances was performed.

## 3. Results

### 3.1. Expression of Gene Lr34 and Lr67 Genes in Analyzed Varieties at Different Time Points

**Figure 2:**
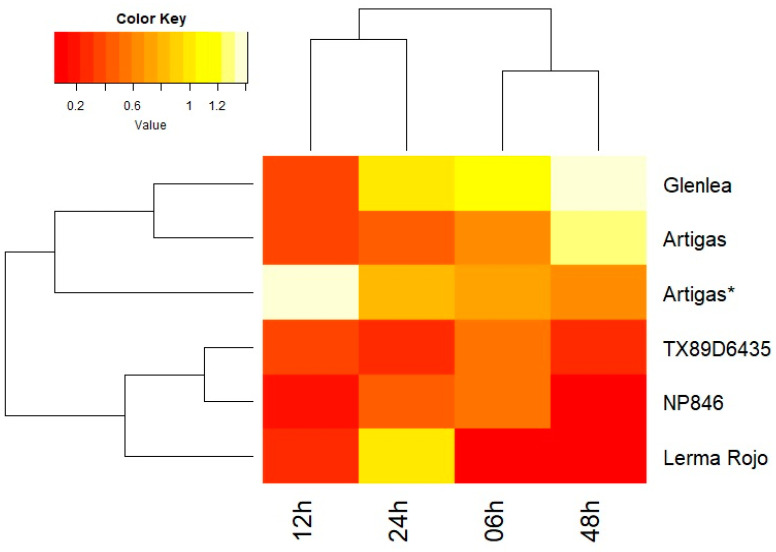
The heatmap diagram shows the expression profiles of the *Lr34* gene obtained using quantitative real-time PCR analysis. The assay included reference varieties of common wheat (Glenlea, Artigas, Artigas*, TX89D6435, NP846 and Lerma Rojo) at four time points (6, 12, 24 and 48 hpi). Red indicates lower relative expression and yellow indicates higher expression.

The response of the *Lr34* gene to inoculation with *P. triticina* was analyzed in four spring (NP846, Glenlea, Artigas, Lerma Rojo) and one winter (TX89D6435) common wheat varieties at five time points (0, 6, 12, 24 and 48 hpi). All varieties except Glenlea revealed a decrease in *Lr34* expression levels immediately after inoculation (6 hpi) (Figure 2). The Glenlea variety also responded in the same way but with a slight delay (12 hpi). Meanwhile, at that time point Artigas and NP486 responded with a further decrease in *Lr34* expression. After 24 h, an increase in *Lr34* expression occurred in all varieties except for TX89D6435, reaching in Glenlea and Lerma Rojo levels similar to that before inoculation. Studied cultivars significantly differed in *Lr34* expression at 48 hpi: in Glenlea and Artigas it reached the maximum values, whereas in the other cultivars it reached the minimum (TX89D6435 and NP846) or very close to the minimum (Lerma Rojo). Observed differences in expression profiles did not follow differences in plant phenology (spring vs. winter). It should be noted that despite significant repression after inoculation, *Lr34* expression did not drop below the level observed in the Artigas* variety (having a susceptibility allele) in all tested time point × genotype combinations except for 6 hpi Lerma Rojo. It appears that the *Lr34* gene is expressed at a relatively high level regardless of inoculation, while after inoculation there is a rapid decrease in expression (realized within 6–12 h). In two lines, this decrease was transient. In Glenlea, Lerma Rojo and NP846, the decrease in expression was statistically significant (*p*-value 0.013–0.048). The lack of statistical support for Artigas and TX89D6435 results from a high variance between biological replicates.

**Figure 3:**
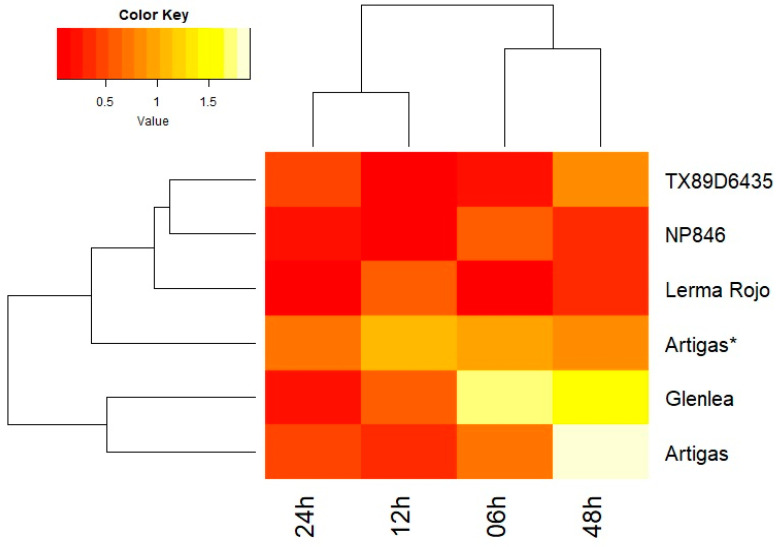
The heatmap diagram shows the expression profiles of the *Lr67* gene obtained using quantitative real-time PCR analysis. The assay included reference varieties of common wheat (TX89D6435, NP846, Lerma Rojo, Artigas*, Glenlea and Artigas) at four time points (6, 12, 24 and 48 hpi). Red indicates lower relative expression and yellow indicates higher gene expression.

The *Lr67* gene, as compared to the *Lr34*, showed a partially different expression pattern also shown by the analysis of variance (Figure 3). In the case of the Artigas, NP846 and TX89D6435 varieties, the expression of the *Lr67* gene decreased immediately after inoculation (6 and 12 hpi) and began to increase after 24 h. In the case of the Lerma Rojo variety, *Lr67* gene expression decreased after inoculation and fluctuated thereafter, while in the Glenlea variety, *Lr67* expression first increased (6 hpi) and then decreased reaching a minimum at 24 hpi, followed by another increase at 48 hpi. The *Lr67* gene in Artigas and Glenlea varieties revealed a similar response to stress like the *Lr34* gene, surpassing in at least one time point after inoculation the level observed in control plants. In the Artigas* line, carrying a susceptible *Lr67* allele, changes in expression levels relative to the first term were not statistically significant. This would imply that only the functional copy of the gene is regulated. Both tested genes are transporters, and as such may have a remarkable effect on the phenotype in terms of the level of accumulation and secretion of the compounds they transport. Interestingly, both genes showed a similar response to inoculation; the correlation between them was 0.675 and that value is statistically significant (*p*-value = 0.0003). On the other hand, considering the difference in expression levels relative to the first term in each line (response to inoculation), those differences were statistically significant for both genes and for the Glenlea, Lerma Rojo, NP846 and TX89D6435 lines, but only for selected time points and only in the direction of a decrease in expression (Supplementary Table S1).

### 3.2. Sanger Sequencing Results

During quantitative PCR analysis of *Lr34* gene expression on the template, differences in melting temperatures (T_m_) were observed between resistant and susceptible varieties (Artigas*), suggesting putative differences in the sequence of the obtained amplicons. Similar observations were made for the *Lr67* gene. Sanger sequencing was performed to verify if there is any polymorphism. The sequence of the *Lr34* gene was confirmed to be complementary to the region 141,646–141,853 bp on reverse-complement reference *Lr34* locus sequence FJ436983.1, spanning exons 7 and 8, 967 bp upstream of the TTC insertion that differentiate resistant and susceptible accessions. The resulting product was identical to the reference sequence with several heterozygous nucleotide positions. The alternative nucleotides present at heterozygous positions which correspond to homologs localized in chromosomes 4A and 7A. For the *Lr67* gene, a PCR product was aligned to the region localized at 1350–1467 bp on reference *Lr67* locus sequence MK425206.1 in 3rd exon, 191 bp downstream of the 1159T/G (Leu387/Val) substitution distinguishing between resistant and susceptible accessions. One heterozygous nucleotide was identified (25C/G), corresponding to homologs present in 4A and 4B chromosomes. Sequences of both PCR products were provided in Supplementary Table S2.

### 3.3. Expression Profiles of miRNAs Associated with the Lr34 Gene

**Figure 4:**
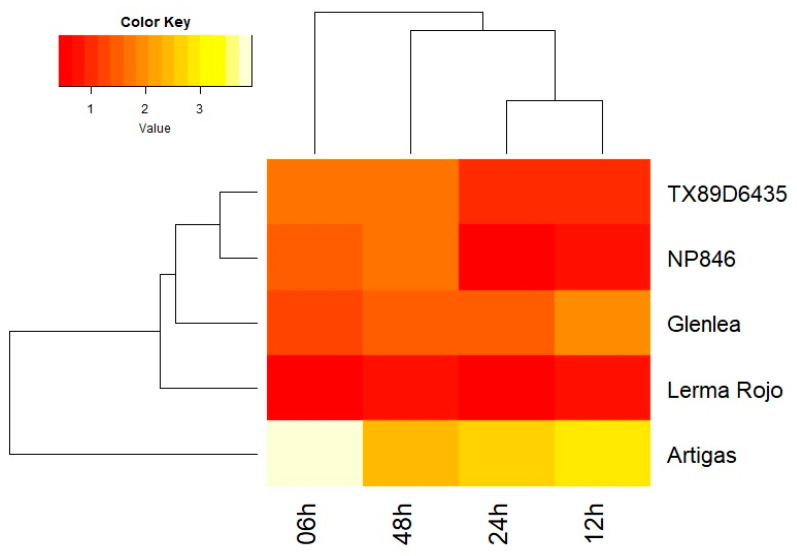
The heatmap shows the expression profiles of tae-miR9653b molecules. The red color indicates downregulation, and yellow indicates upregulation. The assay included reference varieties of common wheat (TX89D6435, NP846, Glenlea, Lerma Rojo and Artigas) at four time points (6, 12, 24 and 48 hpi).

Analysis of miRNA molecules using ddPCR revealed a potential downregulatory link between tae-miR9653b and *Lr34* gene expression in Artigas and Glenlea cultivars (Table 5). In the Artigas variety, an increase of tae-miR9653b copy number immediately after inoculation (6 hpi) was observed, putatively corresponding to a decrease in *Lr34* gene expression. Systematic decline of tae-miR9653b copy number that occurred in further time points correlates with an increase in *Lr34* gene expression observed in that variety. A similar scenario was also revealed in Glenlea. These results indicate that tae-miR9653b may control the degradation of *Lr34* gene transcripts in these two cultivars. Interestingly, the Artigas cultivar revealed the induction of all three studied mRNAs in response to inoculation; however, these changes were only statistically significant in all time points for tae-miR9653b. The comparative data are shown as a heatmap plot for tae-miR9653b expression (Figure 4).

## 4. Discussion

Recent insight into the scientific progress in deciphering of molecular mechanisms underlying wheat resistance to leaf rust led to the conclusion that further search for diverse and durable resistance to leaf rust should harness advanced tools and techniques [2]. Understanding the molecular basis of defense response will allow the development of new strategies for rust resistance in wheat. Even in the presented study, the well-established *Lr34* and *Lr67* genes showed differences in expression profiles between cultivars, despite the presence of the same resistant alleles. It may indicate that there is some variability between studied lines in regulatory pathways acting upstream of these genes. In one of the most recent papers [32], researchers conducted an expression analysis using qRT-PCR after running the *Lr67* transgene into barley to analyze the resistance response of seedlings and adult plants to infection by the leaf rust pathogen. Seedling resistance to the barley-specific diseases *P. hordei* and *Blumeria graminis* f. sp. *hordei* was seen in stable transgenic *Lr67res* barley lines at 24 h postinoculation (hpi), *Lr67res* was two to three times more induced in the *Lr67res* lines following the inoculation of barley plants with *Puccinia hordei.* At 72 hpi, *Lr67res* expression returned to basal levels, and controls did not exhibit induction. Moreover, higher expression of pathogenesis-related (PR) genes and early senescence were both seen in barley plants harboring *Lr67res*.

Genetic expression regulation is one of the factors that determine a plant’s susceptibility or resistance. Nevertheless, it should be noted that such proteins such as ABC transporters may be regulated by multiple signaling pathways and response in different way to different pathogens, including transient downregulation [33]. In our experiments, we revealed that the resistance response of *Lr34* gene in Glenlea and Artigas cultivars to inoculation with *P. triticina* resembled expected scenario for an active antifungal component, i.e., increase of expression after colonization of plant tissue by the fungus. In the work of Bolton et al. (2008), *Lr34* gene expression was analyzed in response to *P. triticina* infection of wheat., and according to the authors, the *Lr34* gene slows disease development, but does not stop pathogen growth [34]. Its resistance phenotype resembles basal plant immunity resulting from recognition of a generic fungal elicitor or pathogen-associated molecular patterns (PAMPs). Leaf tissue was collected at two time points: 3 and 7 days (days postinoculation). The above test was conducted on lines containing *Lr34res*, which showed the induction of several PR genes (pathogenesis-related). *TcLr34* leaves taken at 3 dpi were examined and found significant upregulation of PR-1, PR-2 and PR-3 after infection. This response at the metabolic level of the plant did not persist for 7 dpi; this may explain why the *Lr34* gene is characterized by slowing down the pathogen rather than inhibiting disease development. Researchers’ studies indicate that *Lr34*-induced resistance imposes a high energy demand, which leads to the induction of multiple metabolic reactions to support cellular energy requirements. An abscisic acid (ABA) has been proposed as a candidate molecule that contributes to *Lr34*res-mediated disease resistance [35]. Indeed, the expression of *Lr34* in transgenic barley provides resistance to leaf rust and leads to the accumulation of ABA at the leaf tip and the development of leaf tip necrosis [36,37]. Therefore, upregulation of the *Lr34* gene after infection should be expected as a defense response mechanism.

Recent work [38] has described the interactions of these two APR genes with other *Lr* resistance genes. The researchers compared the effects of *Lr34* and *Lr67* alone, as well as in combination with *Lr13*, *Lr16* or *Lr32*. The *Lr67* and *Lr34* genes significantly reduced the level of severity of rust disease, *Lr34* showed a significant interaction with *Lr13* unlike *Lr67*. Both genes interacted with *Lr16*, and *Lr67* had a significant interaction with *Lr32*. This analysis demonstrates a similar effect of the *Lr67* gene, as observed for *Lr34*, to interact with other resistance genes to achieve better levels of resistance than single resistance genes. The researchers suggest that although *Lr67* is not widely used in agriculture, it may play an important role in disease resistance in future wheat varieties.

In the present study, the expression of candidate miRNA molecules (tae-miR9653b, tae-miR9773 and tae-miR9677b) that could be hypothetically associated with the *Lr34* resistance gene when the plant response to leaf rust disease was analyzed. One of these genes, tae-miR9653b, revealed an expression profile supporting the hypothesis of *Lr34* miRNA dependent decay. Moreover, we conducted preliminary studies with analysis of complementary miRNAs to the *Lr34* and *Lr46* gene [39,40]. Previous studies have already reported that the miRNAs analyzed in this study may be involved in the immune response to both biotic and abiotic stresses. According to studies, tae-miR9773 plays an important role in the biotic stress response in wheat [41]. Kumar et al. (2017) in their work analyzed ten miRNA molecules responsive to leaf rust disease using wheat varieties with the *Lr24* resistance gene (resistant) and varieties lacking the *Lr24* resistance gene (susceptible) [42]. The researchers used stem-loop RT-qPCR to identify and analyze their expression. The researchers reported that the results of their analysis showed differential expression patterns of each miRNA, which may indicate their differential role at different time points after pathogen infection. The maximum expression of two miRNAs, TamiR119 and TamiR256, occurred at 48 hpi while four, TamiR148, TamiR181, TamiR383 and TamiR441, showed a maximum expression at 12 hpi. In contrast, three more miRNA molecules (TamiR218, TamiR337 and TamiR358) showed a maximum expression at 168 hpi, which may indicate their late immune response to leaf rust. As the researchers have pointed out, a significant correlation was observed for all the analyzed miRNA molecules, whose pathogen-induced expression levels were higher in susceptible plants compared to resistant plants, which may indicate an important role for the *Lr24* gene [32]. Li et al. (2017) examined various miRNAs after exposing wheat to phenanthrene, including miR9653b, whose expression was downregulated [41]. In a study on drought stress in common wheat [43], authors considered the role of miRNA molecules on the drought response. The only miRNA suppressed by drought in the drought-tolerant variety was miR9653 (including miR9653a and miR9653b members). In contrast, in the more sensitive wheat variety, the abundance of miR9653b was increased by stress, while the number of miR9653a was unchanged. Unfortunately, there is still limited knowledge about the role of miR9653b in plant pathogen stress responses albeit it was shown that it is positively responsive to *Fusarium graminearum* infection in common wheat lines carrying the 2DL FHB resistance locus [44].

## 5. Conclusions

The presented study showed that the expression of *Lr34* and *Lr67* genes varied at selected time points. After the inoculation, there was a decrease in the level of gene expression in most lines, and then an increase to the level before was observed. The immune system responded most effectively in the Glenlea and Artigas varieties, as 48 h after infection the expression levels of both genes exceeded their baseline expression levels. Based on this study, in the case of the *Lr34* gene, the activation of the immune system in all analyzed varieties was recorded 24 h after inoculation. On the other hand, the *Lr67* gene showed differential expressions within varieties, as shown by the analysis of variance. Finally, the Artigas and Glenlea varieties responded most strongly to stress, as the expression level of the *Lr67* gene 48 h postinoculation, like that of the *Lr34* gene, exceeded the preinoculation baseline. The results indicate that *Lr34* gene expression may be blocked by tae-miR9653b complementary to its sequence in Artigas and Glenlea cultivars. For these two varieties, the expression level of *Lr34* was negatively correlated with the microRNA level of tae-miR9653b, which hypothetically may indicate downregulation. Similar relationships were observed for NP846 and TX89D6435 varieties at almost all time points. These relationships also coincided at some time points for Glenlea (12 hpi and 24 hpi) and Lerma Rojo (24 hpi and 48 hpi) cultivars.

## Figures and Tables

**Table 1:** Name and sequence of designed primers based on *Lr34* and *Lr67* sequences gene.

No.	Gene Name	Primer Sequence (5′-3′)	Product Size (bp)	Amplification Efficiency (E%)	R^2^	T_m_ (°C)
1.	*Lr34*	F GGTAGTAGCAGTTGAAGCR CTCTTCTCATTGCATCCC	110	109.4	0.994	81.0
2.	*Lr67*	F CGCCATCTTCATCTTCTTCR CTGCTTCCACACCTTGTC	118	100	1.000	85.0

**Table 2:** Primer sequences for selected reference genes.

Gene Name	Gene Function/Product	Primer Sequence (5′-3′)	qPCR Efficiency (%)	R^2^	T_m_ (°C)	Size Product (bp)
*TUBβ*	β-Tubulin	F CAAGGAGGTGGACGAGCAGATGR GACTTGACGTTGTTGGGGATCCA	101.1	0.998	82.0	84
*ARF*	ADP-ribosylation factor	F GCTCTCCAACAACATTGCCAACR GCTTCTGCCTGTCACATACGC	93.5	0.999	82.0	165
*RLI*	RNase L inhibitor-like protein	F CGATTCAGAGCAGCGTATTGTTGR AGTTGGTCGGGTCTCTTCTAAATG	-	-	-	242
*EF2-1*	Elongation factor	F CATCAAGCGCATGTCTTCCGR GGTCGACCGTGTTCTTCCAT	100.4	0.997	80.5	90

**Table 3:** Stem-loop primer sequences designed for miRNA reverse transcription reactions and universal primers.

Gene	No.	Primer Name	Stem-Loop Primers Sequence (5′-3′)
*Lr34*	1.	RT miR9653b	GTCGTATCCAGTGCAGGGTCCGAGGTATTCGCACTGGATACGACAGCCTC
	2.	RT miR9773	GTCGTATCCAGTGCAGGGTCCGAGGTATCCGCACTGGATACGACTTCACA
	3.	RT miR9677b	GTCGTATCCAGTGCAGGGTCCGAGGTATCCGCACTGGATACGACGGCCAC
Reference gene	4.	Scrabled short RNA (SCR)	AUAGGCCAUAAGGAGUCUCGGUACGUCUUGUAUG
	5.	SCR-Forward RT	ATAGGCCATAAGGAGTCTCGGTACGTCT

**Table 4:** Primers designed for ddPCR reactions to analyze miRNAs expression.

Gene	No.	Primer Name	Primers Sequence (5′-3′)
*Lr34*	1.	miR9653b F	ACGCAGTGGCCAAGGTCTCTT
	2.	miR9773 F	GGCGCGGTTTGTTTTTATGTTATTT
	3.	miR9677b F	ACTCATCAGGGCGGGGAACAG
	4.	Universal R	CCAGTGCAGGGTCCGAGGTA

**Table 5:** Copy number of tae-miR9653b corresponding to its expression level in the analyzed varieties; tested before inoculation and after 6, 12, 24 and 48 hpi in biological and technical replicates (average value for all replicates).

Common Wheat Varieties	tae-miR9653b Concentration (Number of Copies/µL)				
	0 hpi	6 hpi	12 hpi	24 hpi	48 hpi
Artigas	375	1474	1067	998	900
Glenlea	576	737	1117	845	886
Lerma Rojo	1226	806	852	632	926
NP846	712	821	738	410	1182
TX89D6435	1278	2117	1475	1911	2292

## Data Availability

The datasets generated during and/or analyzed during the current study are available from the corresponding author on reasonable request.

## Supplementary Materials

The following supporting information can be downloaded at: https://www.mdpi.com/article/10.3390/genes14071376/s1, Table S1: Changes in expression levels postinoculation (i.e., relative to the first term) for the *Lr34* and *Lr67* genes; Table S2: PCR product sequences for *Lr34* and *Lr67* genes; Table S3: Change in expression levels after inoculation (i.e., relative to the first term) for reference genes *TUBβ* and *ARF*.
