# Supplementary material for: Expression Profiling of the Slow Rusting Resistance Genes Lr34/Yr18 and Lr67/Yr46 in Common Wheat (Triticum aestivum L.) and Associated miRNAs Patterns
Source: Genes (Basel). 2023 Jun 29;14(7):1376. doi: 10.3390/genes14071376 (PMC10378930; doi:10.3390/genes14071376)
Supplement: Supplementary file 1 [file genes-14-01376-s001.zip › genes-2475248-supplementary.pdf]

Table S1. Changes in expression levels post inoculation (i.e., relative to the first term) for the *Lr34* and *Lr67* genes.

		**Change after inoculation *Lr34***	**Normal distribution (*Lr34*)**	**Equality of variances (*Lr34*)**	**No difference between means (*Lr34*)**	**Correlation between *Lr34* and *Lr67***	**Change after inoculation *Lr67***	**Normal distribution (*Lr67*)**	**Equality of variances (*Lr67*)**	**No difference between means (*Lr67*)**
**Line**	**Time**	T/0h Lr34	Kolmogorov-Smirnov test	Levene's test	Two-tailed t-Student test	0.675191992	T/0h Lr67	Kolmogorov-Smirnov test	Levene's test	Two-tailed t-Student test
**Artigas**	**00h**		0.33063					0.64822		
**Artigas**	**06h**	0.66		0.8877	0.378182		0.73		0.3277	0.478664
**Artigas**	**12h**	0.33		0.7626	0.09917		0.29		0.1986	0.09688
**Artigas**	**24h**	0.49		0.6482	0.160616		0.42		0.5631	0.20285
**Artigas**	**48h**	1.25		0.6301	0.63467		1.90		0.4418	0.068915
**Glenlea**	**00h**		0.46378					0.7998		
**Glenlea**	**06h**	1.06		0.3846	0.909998		1.75		0.3065	0.352663
**Glenlea**	**12h**	0.36		0.3172	0.013023		0.65		0.8707	0.372808
**Glenlea**	**24h**	1.01		0.6651	0.98669		0.28		0.6244	0.049027
**Glenlea**	**48h**	1.40		0.4119	0.750744		1.47		0.44	0.411655
**Lerma Rojo**	**00h**		0.24242					0.64438		
**Lerma Rojo**	**06h**	0.05		0.2972	0.032906		0.13		0.5463	0.020879
**Lerma Rojo**	**12h**	0.23		0.3515	0.062264		0.59		0.9217	0.246334
**Lerma Rojo**	**24h**	1.00		0.5129	0.991322		0.13		0.3339	0.015846
**Lerma Rojo**	**48h**	0.07		0.3566	0.036737		0.40		0.9401	0.102186
**NP846**	**00h**		0.54593					0.2528		
**NP846**	**06h**	0.54		0.4925	0.247717		0.57		0.5177	0.215078
**NP846**	**12h**	0.15		0.2011	0.047787		0.09		0.1792	0.002264
**NP846**	**24h**	0.49		0.3626	0.191111		0.23		0.3396	0.005186
**NP846**	**48h**	0.04		0.1585	0.03256		0.30		0.7344	0.035289
**TX89D6435**	**00h**		0.02799					0.36779		
**TX89D6435**	**06h**	0.53		0.5764	0.602064		0.28		0.3695	0.104686
**TX89D6435**	**12h**	0.33		0.3729	0.442623		0.04		0.1228	0.037423
**TX89D6435**	**24h**	0.32		0.376	0.435676		0.41		0.2006	0.140425
**TX89D6435**	**48h**	0.26		0.4265	0.404337		0.87		0.7883	0.816887
**Artigas***	**00h**		0.96761					0.47516		
**Artigas***	**06h**	0.76		0.2669	0.489588		0.99		0.222	0.982821
**Artigas***	**12h**	1.41		0.5659	0.080655		1.05		0.4515	0.642097
**Artigas***	**24h**	0.78		0.188	0.556859		0.77		0.3577	0.614254
**Artigas***	**48h**	0.67		0.3095	0.241431		0.83		0.1485	0.614593

Table S2. PCR product sequences for *Lr34* and *Lr67* genes.

Gene	Line	Sequences
Lr34	Artigas*	**GGTAGTAGCAGTTGAAGC**TTCAGAGCGAAGCCTACAGACAGATTATATTTTGAAGGTACCCCTTGCCAAAACATCTAAGTTTATGCATAACTTGGTGCTGAAGGCAGATAGGAACAAGATACTAACAGAATCACAATACTGATTTATTTGTAGATCATGGGGCTAGAGATATGCGCAGACACGATGGTTG**GGGATGCAATGAGAAGAG**
	Glenlea	GGTAGTAGCAGTTGAAGCTTCAGAGCGAAGCCTACAGACAGATTATATTTTGAAGGTAaCtCTTGCCAAAACATaTAAGTTTATGCATAACTTGGTGCTGAAGGCAGATAGGAACAAtATACTAACAGAATCACAcTACTGATTTATTTGTAGATCATGGGGCTAGAGAcATGCGCAGACACGATGGTTGGGGATGCAATGAGAAGAG
Lr67	Artigas*	**CGCCATCTTCATCTTCTTC**TCGGCSTGGGTGCTCGTCATGTCCGTCTTCGTGCTCTTCTTCCTCCCGGAGACCAAGAACGTGCCCATCGAGGAGATGACC**GACAAGGTGTGGAAGCAG**
	Glenlea	**CGCCATCTTCATCTTCTTC**TCGGCSTGGGTGCTCGTCATGTCCGTCTTCGTGCTCTTCTTCCTCCCGGAGACCAAGAACGTGCCCATCGAGGAGATGACC**GACAAGGTGTGGAAGCAG**

Table S3. Change in expression levels after inoculation (i.e., relative to the first term) for reference genes *TUBβ* and *ARF*.

		Difference after inoculation *TUBβ*	Change after inoculation *TUBβ*	Normal distribution (*TUBβ*)	Equality of variances (*TUBβ*)	No difference between means (*TUBβ*)	Difference after inoculation *ARF*	Change after inoculation *ARF*	Normal distribution (*ARF*)	Equality of variances (*ARF*)	No difference between means (*ARF*)	No difference between means - both references
**Line**	**Time**	**Cq_T - Cq_00h**	**Cq_T / Cq_00h**	**Kolmogorov-Smirnov test**	**Levene's test**	**Two-tailed t-Student test**	**Cq_T - Cq_00h**	**Cq_T / Cq_00h**	**Kolmogorov-Smirnov test**	**Levene's test**	**Two-tailed t-Student test**	**Two-tailed t-Student test**
**Artigas**	**00h**			0.95454					0.22863			
**Artigas**	**06h**	1.593	1.05431201		0.1626	0.030043	0.960	1.032989691		0.2906	0.223486	0.043641
**Artigas**	**12h**	-0.847	0.971139643		0.7089	0.410666	-1.437	0.950630011		0.4596	0.263805	0.298333
**Artigas**	**24h**	1.633	1.055675491		0.9521	0.076802	1.297	1.044558992		0.7223	0.033396	0.042652
**Artigas**	**48h**	-0.920	0.968639927		0.7187	0.400171	-0.857	0.970561283		0.3809	0.415778	0.376787
**Glenlea**	**00h**			0.6789					0.23878			
**Glenlea**	**06h**	1.487	1.051123338		0.4082	0.050354	0.127	1.004286037		0.8069	0.840002	0.066806
**Glenlea**	**12h**	1.637	1.056281522		0.2962	0.034952	0.420	1.014211595		0.5766	0.504937	0.021232
**Glenlea**	**24h**	1.377	1.047340669		0.8298	0.158302	2.453	1.08301376		0.4407	0.11911	0.099561
**Glenlea**	**48h**	-0.880	0.969738652		0.4112	0.474999	-1.567	0.946988495		0.2305	0.190415	0.308638
**Lerma Rojo**	**00h**			0.86625					0.62449			
**Lerma Rojo**	**06h**	1.057	1.038114705		0.7891	0.064677	2.130	1.07596291		0.4908	0.555619	0.425315
**Lerma Rojo**	**12h**	0.723	1.026091139		0.8683	0.243417	0.993	1.035425583		0.5303	0.265889	0.179071
**Lerma Rojo**	**24h**	3.157	1.113863172		0.9314	0.002255	3.110	1.110912981		0.3869	0.012561	0.004638
**Lerma Rojo**	**48h**	-1.030	0.96284718		0.7944	0.121469	-1.130	0.959700428		0.3783	0.193768	0.1482
**NP846**	**00h**			0.85829					0.89651			
**NP846**	**06h**	1.983	1.069925961		0.272	0.007126	1.097	1.039758308		0.4756	0.075348	0.008003
**NP846**	**12h**	1.230	1.043365848		0.4395	0.013219	1.830	1.066344411		0.7153	0.022311	0.015284
**NP846**	**24h**	2.243	1.079092725		0.2413	0.048836	3.767	1.136555891		0.604	0.018252	0.025251
**NP846**	**48h**	-1.523	0.946292161		0.2628	0.200372	-0.713	0.974138973		0.729	0.250815	0.184133
**TX 89D6435**	**00h**			0.99762					0.87176			
**TX 89D6435**	**06h**	3.440	1.128855038		0.8188	0.013352	4.010	1.154210999		0.9203	0.116369	0.055147
**TX 89D6435**	**12h**	1.290	1.048320639		0.3196	0.119505	1.480	1.05691578		0.1836	0.348178	0.234443
**TX 89D6435**	**24h**	2.690	1.100761643		0.8005	0.028141	3.527	1.135623638		0.2058	0.061867	0.041902
**TX 89D6435**	**48h**	1.220	1.045698589		1	0.247515	2.333	1.089732086		0.7659	0.251491	0.234053
